# The Dental Colleges

**Published:** 1874-10

**Authors:** 


### THE DENTAL COLLEGES.

The time for the opening of the regular courses in the various dental colleges in the country is near at hand, and the indications from each are, that their classes will be as large, if not larger, the coming session than heretofore. This is certainly desirable with some at least; but securing the largest possible number of students is not the only thing to which special attention should be given. Enlargement of the curriculum and more systematic methods of giving instruction is certainly worthy of attention. Greater thoroughness in the branches studied should be insisted upon. The most perfect methods of practice should be presented by the teachers, and a thorough mastery of them by the student be insisted upon. In the clinical department, a graduated, systematic course should be marked out, and every student should be required to make thorough work in each and every step of the course. By adhering rigidly to such a course much greater proficiency will be obtained. As thoroughness is attained in one direction it will lead to the same result in other branches. Another matter to which teachers in our colleges ought to give special attention is the eultivation of those under their charge in a just and proper appreciation of professional character.

It is true that the presentation and pressing the truth, in this direction, will not make of an inbred charlatan and trickster an upright, honorable and dignified professional gentleman; yet many enter our colleges who, so far as professional character is concerned, are in the forming stage, and are susceptible of direction in either the right or wrong course. Let every teacher see to it, that he performs his duty in this respect.

Another particular in which our colleges should exercise more care, is the selection of students.

Doubtless, many who are totally without natural ability or adaptation for a professional life of any kind, and especially dentistry, through ignorance of what is involved in its successful practice, seek to enter it.

There is a temptation for a college faculty to encourage such or, at least, not to discourage them. The position has been taken that, the teachers have nothing to do with such things, maintaining that their only duty is simply to teach those who present themselves. If the only aim is to secure the largest possible number of students, and then pass over with them a mere routine course of instruction, with no thought for future results, then the position and reasoning may be good.

No man who has the good of his profession at heart, or a true regard for the welfare of his fellow men, will rest satisfied with such a view.

Our dental colleges should exercise a discriminating care in reference to those they admit to their classes.

It has been said that none can be excluded. The falsity of this is clearly shown in the custom of every literary and scientific institution, of any pretensions in the country.

We shall be glad to see students of dentistry admitted to our colleges only by the passage of a proper examination, upon such a grade as might be agreed upon by all or, at least, a large majority of the dental colleges in the country. The plan that has thus far been practiced and encouraged is one of the dire embarrassments that environ the subject of dental education at the present time. May a change for the better upon be inaugurated,

